# The Peak–End Rule and Retrospective Emotional Valence in Digital Learning Tasks: Evidence from a Word-Learning App

**DOI:** 10.3390/bs16050779

**Published:** 2026-05-14

**Authors:** Wei Xie, Zhitao Li

**Affiliations:** School of Digital Technology and Creative Design, Jiangnan University, Wuxi 214122, China; 6230306028@stu.jiangnan.edu.cn

**Keywords:** peak–end rule, retrospective emotional valence, emotional design, human–computer interaction, digital learning

## Abstract

The peak–end rule proposes that retrospective evaluations depend on the emotional peak and the end of an experience rather than on its duration. Two short, controlled vocabulary-learning experiments tested whether optimizing these moments improves retrospective emotional valence. Study 1 (*N* = 32) manipulated task length (4 vs. 8 words). Retrospective emotional valence did not differ significantly between groups (*p* = 0.459, *d* = 0.27), a result consistent with duration neglect under this short task–episode manipulation but not a strong test of pure temporal duration neglect. Retrospective emotional valence correlated more strongly with the peak–end mean than with the mean of reconstructed page-level ratings (*r* = 0.761 vs. *r* = 0.314; Steiger’s *Z* = 3.03, *p* = 0.002). Study 2 (*N* = 56) used a 2 × 2 design to optimize the candidate peak-related completion page and the structurally defined end check-in page through color and anthropomorphic graphics. Both peak (*ηp*^2^ = 0.18) and end (*ηp*^2^ = 0.22) optimization enhanced retrospective emotional valence, with a significant non-additive interaction (*ηp*^2^ = 0.09): the effect of optimizing one node was reduced when the other node had already been optimized. For learning accuracy, the main effect of peak optimization was significant (*F*(1, 52) = 4.44, *p* = 0.040), but only the combined peak-and-end optimization significantly outperformed the control condition (*p* = 0.041, *d* = 1.11); neither single-optimization condition significantly differed from the control condition after correction. The findings provide preliminary evidence for a peak–end-consistent evaluation pattern in brief, controlled vocabulary-learning tasks, identify a non-additive interaction in peak–end optimization, and offer guidance for designing key interactive moments within similarly short, task-based learning episodes.

## 1. Introduction

### 1.1. Concept and Core Characteristics of the Peak–End Rule

How do people evaluate an experience after it ends? This question matters because retrospective evaluations shape future choices. In cognitive psychology, the peak–end rule explains how such judgments are formed. It proposes that an individual’s overall retrospective evaluation of a past event is determined primarily by the moment of greatest pleasure or pain (the peak) and the feeling at the moment the experience ends (the end), rather than a simple average of momentary utilities over time (Fredrickson & Kahneman, 1993; Kahneman et al., 1993). This rule reflects the dual-system character of human memory: although the experiencing self undergoes affect in real time, the remembering self relies heavily on these two anchors to construct retrospective evaluations, producing the systematic deviation known as the experience–memory gap (Fredrickson, 2000; Kahneman & Riis, 2005). The rule comprises two core cognitive principles. First, peak–end dominance means that individuals disproportionately weight representative key moments during memory construction (Kahneman et al., 1993). Second, duration neglect means that the actual duration of an experience has a limited influence on its retrospective evaluation. Evidence from diverse domains, including clinical pain (Redelmeier & Kahneman, 1996) and aversive sounds (Schreiber & Kahneman, 2000), shows that extending an experience does not necessarily change its evaluation if the peak and end remain unchanged. Although duration may influence evaluations if it becomes a salient negative feature (Ariely & Carmon, 2000), the primary implication is that human memory construction prioritizes affective snapshots over cumulative temporal utility.

### 1.2. The Peak–End Rule in Human–Computer Interaction

In human–computer interaction (HCI), the peak–end rule explains how users form impressions of digital systems. Research confirms its robustness across diverse interfaces: users’ overall evaluations depend on peak and end moments in interactive products (Hassenzahl & Ullrich, 2007), slider tasks (Cockburn et al., 2017), online video viewing (Ryu & Kim, 2021), mobile commerce (Li et al., 2023), and immersive environments such as VR (Warsinke et al., 2024). Recent work has also applied this rule to educational app design, identifying peak and end moments in online learning journeys to improve student experience (Zeng et al., 2023). Together, these findings support a shift in UX design from intuition-driven optimization to cognitively informed design.

However, most existing studies have used passive-consumption tasks or tasks with low cognitive demands. Cockburn et al. (2017) used simple motor slider tasks without semantic processing. Ryu and Kim (2021) examined passive video viewing rather than active knowledge construction. Li et al. (2023) studied e-commerce interactions driven by instrumental goals rather than cognitively effortful learning. Consequently, whether peak–end patterns also appear during brief but cognitively active learning episodes remains insufficiently tested.

This issue is relevant to vocabulary-learning applications because learners must encode word meanings and update working memory while interacting with the interface. These processes impose cognitive load (Mayer & Fiorella, 2021). Unlike passive viewing, in which affective fluctuations may dominate, learning tasks allocate limited cognitive resources to knowledge acquisition. Whether the remembering self still relies on peak–end affective anchors when the experiencing self is engaged in cognitive processing remains an open question.

Prior research has examined the peak–end rule in online learning (Brom et al., 2017; Zeng et al., 2023), and other studies have shown that emotional design features can enhance multimedia learning outcomes (Wang et al., 2023). However, these studies did not systematically manipulate peak and end nodes under controlled conditions. The studies by Brom and Zeng were largely theoretical or correlational, whereas Wang et al. experimentally tested emotional design elements rather than the specific effects of peak and end moments.

### 1.3. Emotional Intervention Methods in Interactive Interfaces

In HCI, interface-elicited emotions influence cognitive processing, interaction outcomes, and system evaluation (Brave & Nass, 2007; Norman, 2004). Tractinsky et al. (2000) reported a strong correlation between interface aesthetics and perceived usability, suggesting that emotional experience contributes to system evaluations. Emotional design thus uses interface elements to intentionally improve users’ affective states and, in turn, their experience or performance.

Prior research has identified two main approaches to emotion induction in interactive interfaces:

External emotion induction elicits an affective state before an interaction task using materials unrelated to the interface content, such as films, music, or emotional corpora (Knörzer et al., 2016; Park et al., 2015; Plass et al., 2014). This method is easy to implement and has clear effects, but the induced emotions are difficult to sustain throughout the entire interaction process. In longer tasks, emotions may decay during later stages, leading to uneven intervention effects (Knörzer et al., 2016).

Internal emotional design induces emotion during interaction through visual aesthetics and feedback. For example, warm, highly saturated colors (Plass et al., 2014) and more anthropomorphic graphics (Mayer & Estrella, 2014) can increase an interface’s emotional appeal and influence users’ affective states throughout the task. In a study of webpage complexity, Geissler et al. (2013) found that visual designs with moderate complexity, including gradient colors, improved users’ attitudes and emotional experiences. Compared with external induction, internal design synchronizes emotion induction with the interaction process, allowing the intervention to persist throughout the task. This feature makes it suitable for fine-grained manipulation at specific task nodes.

Color and anthropomorphic graphics are two widely used elements of internal emotional design. Warm, saturated colors can attract attention and elicit positive affect, such as pleasure and excitement (Elliot & Maier, 2014). Gradient colors are often perceived as harmonious and fluent (Schloss & Palmer, 2011). In interface design, the judicious use of color can significantly enhance users’ emotional experience and satisfaction (Cyr et al., 2010). Anthropomorphic design gives non-human objects human characteristics and can activate users’ social cognitive schemas (Epley et al., 2007). According to the media equation theory (Reeves & Nass, 1996), people unconsciously apply social interaction rules to computers and media. Thus, anthropomorphic interface elements, such as cute characters, facial expressions, and dialog bubbles, can trigger social responses, create familiarity and connection, and induce positive emotions (Sundar et al., 2016). Preliminary research also suggests that color and anthropomorphic elements may produce additive emotional enhancement when used together (Kim & Sundar, 2012).

Internal emotional design, therefore, fits the peak–end framework because it can enhance affect at specific nodes in an interaction sequence. In this study, color and anthropomorphic graphics were used to optimize the identified candidate peak page and the end page. A 2 × 2 design tested the separate and joint effects of these optimizations on retrospective emotional valence.

### 1.4. Research Gaps and the Proposal of This Study

Although the peak–end rule is well established in passive or low-load tasks, its applicability to complex digital learning contexts remains uncertain. The literature lacks systematic evidence on whether the peak–end heuristic operates under the cognitive demands of learning. In addition, existing peak–end design studies in this area are mostly correlational or post hoc and do not experimentally manipulate visual nodes to establish causal effects on retrospective evaluation or learning. To address these gaps, the present study used two experiments to examine whether peak–end-consistent evaluation patterns emerge in brief vocabulary-learning tasks and whether targeted visual optimization of selected pages improves retrospective evaluation. Specifically, we asked: (1) In tasks requiring semantic processing, does a peak–end pattern emerge, and does task length moderate this pattern? (2) Can inducing positive emotions through visual stimuli on the candidate peak-related page and the structurally defined end page enhance users’ retrospective emotional valence and word-learning accuracy? (3) Do peak and end optimizations produce a simple additive effect, or do they interact in a more complex manner?

**Figure 1:**
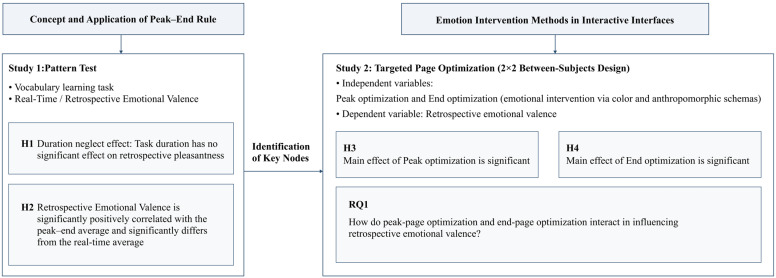
Overview of the two-study research design.

Figure 1 summarizes the two-study research design.

Study 1 examined whether the evaluation pattern observed in vocabulary-learning tasks was consistent with the peak–end rule, focusing on duration neglect and the relative salience of peak and end moments. The specific hypotheses were as follows:

**H1.**  *Under the present word-count-based task configuration, retrospective emotional valence will not differ significantly between the short-task and long-task conditions.*

**H2.**  *Retrospective emotional valence will be more strongly associated with the peak–end average than with the mean of reconstructed page-level ratings.*

Building on Study 1, Study 2 used a 2 × 2 between-subjects design to test the main and interaction effects of visual optimization at the candidate peak and end pages on retrospective emotional valence. The hypotheses were as follows:

**H3.**  *Peak-page optimization will be associated with higher retrospective emotional valence than no peak-page optimization.*

**H4.**  *End-page optimization will be associated with higher retrospective emotional valence than no end-page optimization.*

Because the joint influence of these two nodes on user experience is exploratory, we posed the following research question rather than a directional hypothesis:

**RQ1.**  *How do peak-page optimization and end-page optimization interact in influencing retrospective emotional valence?*

## 2. Materials and Methods

### 2.1. Experimental Materials

#### 2.1.1. Task Selection and Manipulation of Task Length

To preserve a realistic interaction flow while maintaining experimental control, the study used MaiMemo, a commercially available vocabulary-learning application in China, as the stimulus platform. Published technical work associated with the MaiMemo team indicates that the platform has been used for spaced-repetition scheduling research and large-scale memory-behavior logging (Su et al., 2023; Ye et al., 2022). The experimental task used a simplified ‘Learning—Completion—Check-in’ flow that captured a brief learning episode from entry to completion and sign-in.

Task length was operationalized as the number of words learned: the short-task group learned 4 words, and the long-task group learned 8 words. This manipulation necessarily covaried with cognitive load and exposure frequency, reflecting the ecological reality of digital learning, where longer sessions typically involve more material. The present design, therefore, tests the peak–end rule at the task–episode level, where duration and workload covary, rather than isolating pure temporal duration. Pilot observations indicated that learners who set a daily target of 100 words completed a session in approximately 38 min on average. On this basis, the 4-word and 8-word tasks were expected to last approximately 1.6 and 3.2 min, respectively. Although shorter than naturalistic daily learning sessions, this duration was deliberately constrained to balance cognitive engagement against participant fatigue in a controlled laboratory setting.

Accordingly, participants in the short-task group learned 4 words, whereas participants in the long-task group learned 8 words. This design increased the number of cognitive processing episodes and was expected to produce a longer task length, while also introducing differences in workload, repeated exposure, and task progression. Although this confound limits the ability to attribute the null effect solely to duration neglect, it enhances the ecological validity of the manipulation for digital learning contexts.

#### 2.1.2. Stimulus Materials

To control extraneous variance, especially differences in prior vocabulary knowledge, all experimental words were selected from the standardized vocabulary database of the Common European Framework of Reference for Languages (CEFR). The CEFR is an internationally used framework for describing language proficiency across six levels, from A1 to C2 (Council of Europe et al., 2001). Because participants were Chinese non-language-major undergraduates whose English proficiency was expected to begin around CEFR B1, materials were drawn from B1, B2, C1, and C2. This range covered vocabulary demands from foundational to advanced levels that were relevant to the target user group. Schmalz and Brutti (2022) also supported the semantic alignment between CEFR-graded vocabulary and learner proficiency using BERT embeddings.

A stratified random sampling procedure was used. Entries from B1, B2, C1, and C2 were extracted to construct the candidate pool. The short-task group received one word from each level (4 words total), whereas the long-task group received two words from each level (8 words total). This cross-level sampling maintained a comparable distribution of word difficulty between groups while creating a clear task-length contrast.

#### 2.1.3. Difficulty Rating and Material Validation

Material difficulty was validated in a pre-test. Ten words were randomly selected from each CEFR level (B1, B2, C1, and C2), yielding an initial pool of 40 words. Independent raters from the target population (non-English-major undergraduates) rated each word on a 7-point scale (1 = very easy, 7 = very difficult). Words were presented in randomized order under standardized conditions.

An a priori G*Power analysis (version 3.1.9.7) was conducted. Because no direct empirical prior was available for this material-validation task, a medium effect size (f = 0.25), power of 0.80, and four repeated-measures levels were specified following conventional standards (Cohen, 1988). The minimum required sample size was 24; 30 students from the target population completed the rating task.

A one-way repeated-measures ANOVA tested whether perceived difficulty differed across CEFR levels. Sphericity was satisfied (all *p*s > 0.05). Mean perceived difficulty differed significantly across the four levels, *F*(3, 87) = 54.25, *p* < 0.001, *ηp*^2^ = 0.625. Mean ratings increased monotonically with CEFR level (B1: *M* = 2.95, *SD* = 1.02; B2: *M* = 4.14, *SD* = 1.69; C1: *M* = 5.03, *SD* = 1.54; C2: *M* = 5.91, *SD* = 1.32). Bonferroni-corrected post hoc comparisons showed significant differences between all adjacent levels (all *p*s < 0.001), supporting the intended difficulty gradient.

Within-level homogeneity was examined using Cronbach’s *α* for each CEFR level. As shown in Table 1, reliability was high for B1 (*α* = 0.89), B2 (*α* = 0.95), C1 (*α* = 0.94), and C2 (*α* = 0.91), all exceeding the conventional 0.70 threshold. These values indicate that words within each level were sufficiently consistent in perceived difficulty for sampling.

Based on the pre-test, the two most difficult words from each CEFR level were selected as formal materials. Because within-level difficulty was homogeneous, any two words could represent their respective tier. Selecting more difficult words amplified the difficulty gradient between levels, making processing-related differences in cognitive load more detectable and strengthening the experimental manipulation. To mitigate the risk of frustration, the total task length was capped at 8 words, prioritizing brief but meaningful semantic processing over a longer duration while still inducing a measurable time difference. Thus, the short-task group learned one word from each level (4 words total), and the long-task group learned two words from each level (8 words total), maintaining a comparable difficulty distribution across conditions.

### 2.2. Participants

Participants were recruited from non-language-major undergraduates at Jiangnan University, matching the core user demographic of the word-learning application: university students aged 18–25 years. Inclusion criteria were as follows: (1) normal or corrected-to-normal vision, without color blindness or color weakness; (2) proficiency in smartphone use; (3) no prior participation in similar experiments; (4) normal memory function without any memory impairment; and (5) no prior experience with the MaiMemo app, to ensure interface novelty and control for familiarity bias. This study was conducted in accordance with the Declaration of Helsinki and approved by the Ethics Committee of Jiangnan University (protocol code: JNU202403RB139; date of approval: 1 March 2024). All participants provided written informed consent before the experiment and received compensation after completion.

Sample sizes were determined through a priori G*Power analyses (version 3.1.9.7). Because the peak–end rule has rarely been tested in digital learning contexts, effect-size assumptions followed conventional standards for behavioral and HCI research (Cohen, 1988), consistent with prior work on interface-level visual manipulations and retrospective evaluations (e.g., Cockburn et al., 2017; Li et al., 2023) and on emotional design in multimedia learning (Plass et al., 2014).

For Study 1, *α* = 0.05 and power = 0.80 indicated a minimum of 16 participants per group. Thirty-two students were recruited (*M*_age_ = 20.4 years, *SD* = 1.31; 17 females, 15 males) and randomly assigned to the short-task group (*n* = 16) or the long-task group (*n* = 16). The two groups showed no significant differences in gender distribution (short-task group: 7 males, 9 females; long-task group: 8 males, 8 females) or age (short-task group: *M* = 20.3, *SD* = 1.4; long-task group: *M* = 20.5, *SD* = 1.2). Prior knowledge was assumed to be balanced through random assignment.

Study 2 used a 2 × 2 between-subjects factorial design with peak optimization (present vs. absent) and end optimization (present vs. absent). A G*Power analysis indicated a minimum of 13 participants per group. Fourteen participants were recruited per condition, yielding 56 participants (28 males, 28 females; *M*_age_ = 20.6 years, *SD* = 1.2). None had participated in the preliminary optimization test. The four groups did not differ significantly in gender (7 males and 7 females per group), age (range: 20.4–20.8 years, SDs = 1.1–1.3), or prior knowledge.

### 2.3. Experimental Methods and Measures

#### 2.3.1. Experimental Design

The research comprised two controlled laboratory experiments. Study 1 examined whether the evaluation pattern in a brief vocabulary-learning episode was consistent with peak–end assumptions. Study 2 tested whether visual optimization of the candidate peak-related page and end page affected retrospective emotional valence and word-learning accuracy.

Study 1 adopted a single-factor, between-subjects design with two levels (short vs. long task). Its primary aim was to test whether the evaluation pattern aligned with two core assumptions of the peak–end rule: duration neglect and the relative prominence of peak and end moments. Task length was manipulated by varying the number of words learned: 4 words in the short-task group and 8 words in the long-task group. The main dependent variable was retrospective emotional valence. Page-level emotional valence was also measured by replaying the screen recording immediately after the task; this replay produced reconstructed ratings rather than uninterrupted online affect. A total of 32 university students were randomly assigned to the short-task group (*n* = 16) or the long-task group (*n* = 16), with no significant differences in gender, age, or prior knowledge. The experimental task simulated the core flow of a real vocabulary-learning app—Learning, Completion, and Check-in—and constituted a brief, controlled episode: participants completed word learning on a smartphone in a preset order, then performed a final check-in.

Study 2 used a 2 × 2 between-subjects design to examine the main and interaction effects of visual optimization at the candidate peak-related page and the end page on retrospective emotional valence. Independent variables were peak optimization (with vs. without) and end optimization (with vs. without). Peak optimization applied the selected visual package to the task-completion page; this page had been identified in Study 1 as a theoretically plausible and empirically suggested candidate peak. End optimization applied the same package to the check-in page, which served as the structurally defined end page. The visual package was validated through a preliminary manipulation check with an independent, non-overlapping sample (*N* = 30) separate from the main experiment (*N* = 56), eliminating potential carryover and familiarity effects. The preliminary test served strictly as a manipulation check to confirm the emotional efficacy of the pre-determined design variations, not as an exploratory study to derive hypotheses. The primary dependent variable was retrospective emotional valence; page-level valence ratings were also collected via post-task replay, yielding reconstructed page-level evaluations. As a secondary behavioral measure, word-learning accuracy (the proportion of correctly recalled words in a post-task test) was recorded to explore whether interface optimization influenced learning outcomes in this brief setting. A total of 56 university students were randomly assigned to four groups (*n* = 14 each), balanced on demographic variables and prior knowledge. All participants completed an 8-word learning task. Depending on group assignment, they received optimized or unoptimized versions of the candidate peak page and the end page, while all other pages remained unchanged. The experimental procedure was consistent with Study 1, and retrospective emotional assessment was conducted immediately after task completion.

#### 2.3.2. Experimental Procedure

The experiment was conducted in a controlled indoor setting. Participants sat facing a smartphone fixed on a desktop stand, surrounded by opaque baffles that reduced visual distractions. The experimenter managed the procedure from the opposite side of the desk, and a behavior recorder was positioned diagonally behind the participant. The recording supported standardized replay and procedure verification only; no separately coded behavioral data were extracted for inferential analysis. Participants could not observe the experimenter or the recording equipment during the task, reducing expectancy and social-desirability effects.

The procedure included three phases: preparation, formal experiment, and post-test data collection.

Preparation phase: Participants signed the informed consent form, completed a registration form and a demographic pre-test questionnaire, and were guided to the operating position. The experimenter explained the task, confirmed that the equipment was functioning, and asked participants to read the smartphone instruction: “Please complete the given word-learning task and perform the check-in.” After confirming comprehension, the experimenter started the task and began video recording.

Formal experiment phase: After the start command, participants tapped the screen and entered the word-learning page automatically. They then completed the word-learning task according to the preset flow: the short-task group learned 4 words, and the long-task group learned 8 words. During the task, the researchers observed the operations without intervening. Immediately after task completion, the researcher stopped the recording, saved the operation video, and recorded the task length.

Post-test data collection phase: After the task ended, the experimenter replayed the screen video at normal speed and asked participants to rate their emotional valence page by page. To avoid interrupting cognitive flow, we obtained reconstructed page-level ratings via immediate replay rather than online affect. Participants then rated their overall retrospective emotional valence. The experimenter checked the questionnaires for completeness and organized the data by experimental group and participant number.

**Figure 2:**
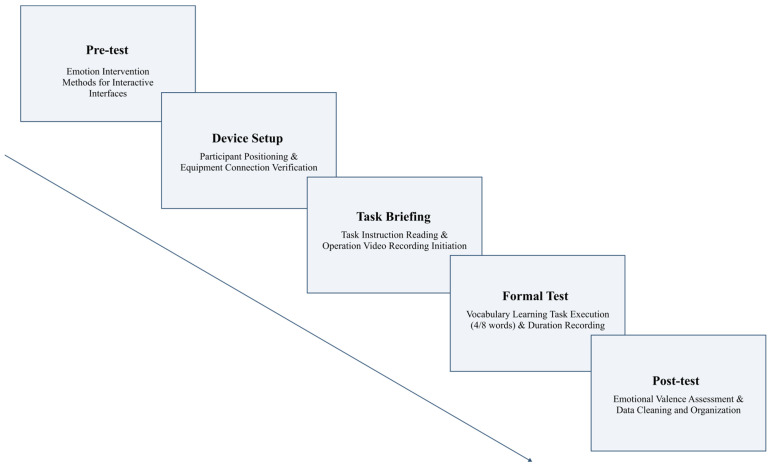
Flowchart of the experimental procedure.

The specific experimental procedure is illustrated in Figure 2.

#### 2.3.3. Experimental Measures

Subjective emotional valence was measured with the Self-Assessment Manikin (SAM) in Study 1 and a Visual Analog Scale (VAS)-based continuous valence procedure in Study 2. The two studies addressed different research questions and were analyzed independently; therefore, absolute valence scores were not compared across studies. Study 1 used the standardized SAM scale to examine overall retrospective evaluation patterns. By contrast, Study 2 assessed the impact of visual design optimizations on emotional valence; these interventions were expected to produce relatively subtle differences across conditions. Compared with discrete scales, the continuous VAS provides higher measurement resolution and sensitivity, making it better able to detect fine-grained valence differences across optimization conditions.

The SAM scale was developed by Bradley and Lang (1994) as a standardized non-verbal pictorial tool for measuring three dimensions of subjective feeling: emotional valence, arousal, and dominance. Study 1 selected the valence dimension as the indicator of participants’ retrospective emotional valence. Russell’s (1980) circumplex model conceptualizes affect in terms of valence and arousal. In foundational peak–end research, however, the peak is operationalized as the moment of highest or lowest valence—the most pleasant or unpleasant instant—rather than as the moment of maximum arousal (Kahneman et al., 1993). The rule primarily predicts retrospective hedonic preferences based on valence direction. Regarding arousal, Strijbosch et al. (2019) found that, in complex heterogeneous experiences, retrospective evaluations were better predicted by average arousal across the whole experience than by arousal at peak–end moments alone. Accordingly, the present study tested the traditional valence-based assumption of the peak–end rule and, consistent with HCI conventions (Cockburn et al., 2017; Hassenzahl & Ullrich, 2007), measured emotional valence only. This choice limits the study’s ability to examine arousal-specific contributions to peak–end patterns. The SAM valence scale uses a 9-point format (1 = very unpleasant, 9 = very pleasant), and participants selected the score that best corresponded to their overall feeling. This tool has been widely used in prior HCI and user experience research and has demonstrated good reliability and validity.

Study 2 employed a VAS-based continuous valence rating procedure informed by the SAM valence framework. This procedure assessed two indicators: participants’ overall retrospective emotional valence after task completion and reconstructed page-level valence ratings obtained through immediate task replay. VAS was first used for pain assessment by Hayes and Patterson in 1921 and was later applied to emotion measurement. Its continuous format provides greater sensitivity to subtle subjective changes than discrete Likert scales (Grant et al., 1999). Because the visual optimizations were expected to produce subtle differences, the VAS format was used to increase measurement resolution for retrospective and reconstructed valence ratings within Study 2. Following Reips and Funke (2008), participants used an online slider ranging from −4 (very unpleasant) to 4 (very pleasant), with 0 as neutral; values were recorded to two decimal places. SAM- and VAS-based procedures have been used in prior studies of emotional responses (Barratt et al., 2016; Cao et al., 2024; Ruef & Levenson, 2007). Although both tools measure emotional valence, they are not psychometrically equivalent, and no cross-study calibration was performed. Because all analyses were conducted within each study rather than through cross-study comparisons of absolute scores, the lack of scale equivalence does not compromise the interpretation of the findings.

The detailed items for these scales are provided in Appendix A and Appendix B.

## 3. Results

### 3.1. Study 1

#### 3.1.1. Manipulation Check

The manipulation check verified that the task-length manipulation produced the intended difference in completion time. As shown in Table 2, an independent-samples *t*-test confirmed that the long-task group took significantly longer to complete the task (*M* = 114.94 s, *SD* = 46.79) than the short-task group (*M* = 60.50 s, *SD* = 21.48). Because Levene’s test indicated unequal variances, *F*(1, 30) = 11.17, *p* = 0.002, Welch’s *t*-test was used. The difference was significant, *t*(21.06) = −4.23, *p* < 0.001, 95% CI [−81.20, −27.67], Cohen’s *d* = −1.50, 95% CI [−2.27, −0.70], indicating a large effect. These results confirmed that the manipulation produced a substantial difference in actual task completion time.

Perceived duration was not directly measured, which limits the strict validation of subjective duration neglect. Nevertheless, this approach is consistent with foundational peak–end studies that inferred duration neglect from objective metrics such as procedure time, sound exposure length, or number of interaction steps (Cockburn et al., 2017; Redelmeier & Kahneman, 1996; Schreiber & Kahneman, 2000). Given the cognitive demands of sequentially processing each word, doubling the objective duration and cognitive episodes (from 4 to 8 words) would have been subjectively salient. Nonetheless, the absence of a direct measure of perceived duration limits strict validation of the subjective dimension of duration neglect.

#### 3.1.2. Test of Time Duration Neglect

To test H1, we compared retrospective emotional valence between the short-task and long-task groups. Consistent with duration neglect, the two groups did not differ significantly despite a substantial difference in completion time.

A Shapiro–Wilk test indicated that retrospective emotional valence ratings were normally distributed in both groups, satisfying the normality assumption for an independent-samples *t*-test. The short-task group (*n* = 16) reported a mean valence of 7.00 (*SD* = 0.89), and the long-task group (*n* = 16) reported a mean of 6.69 (*SD* = 1.40). Levene’s test indicated unequal variances, *F*(1, 30) = 6.02, *p* = 0.020; therefore, Welch’s *t*-test was used. The difference was not significant, *t*(25.49) = 0.75, *p* = 0.459, Cohen’s *d* = 0.27, 95% CI [−0.43, 0.96]. The small effect size and confidence interval crossing zero indicate no statistically reliable difference between groups. Table 3 presents the data.

These results show that the long-task group spent significantly more time on the task, but retrospective emotional valence did not differ significantly between groups. This pattern is consistent with duration neglect under the present short task–episode manipulation. However, because word count also changed cognitive load, exposure frequency, and task progression, the null effect cannot be attributed to pure temporal duration neglect. Other explanations remain possible, including low statistical power, insufficient temporal contrast, compensatory effects between fatigue and achievement, or characteristics of the selected vocabulary items. Consequently, this result should be treated as preliminary and limited to the present manipulation.

Furthermore, retrospective emotional valence ratings in both the short-task group (*M* = 7.00) and the long-task group (*M* = 6.69) were well above the midpoint of the nine-point scale, empirically indicating that the overall task experience remained predominantly positive rather than frustrating. This suggests that the difficulty manipulation induced cognitive challenge without producing excessive overload.

#### 3.1.3. Test of the Peak–End Pattern

H2 predicted that retrospective emotional valence would correlate more strongly with the peak–end average than with the mean of reconstructed page-level ratings. Dependent correlations were compared using Steiger’s *Z* test, supplemented with bias-corrected and accelerated (BCa) bootstrap 95% confidence intervals (10,000 resamples) to ensure robust inference given the modest sample size (*N* = 32, *n* = 16 per condition). Results are summarized in Table 4.

In the overall sample (*N* = 32), retrospective emotional valence correlated strongly with the peak–end average (PE) (*r* = 0.761, *p* < 0.001) and non-significantly with the mean of reconstructed page-level ratings (Average) (*r* = 0.314, *p* = 0.080). The difference was significant, Steiger’s *Z* = 3.03, *p* = 0.002, BCa 95% CI [0.147, 0.747]. The peak–end average accounted for 57.9% of the variance in retrospective emotional valence (*R*^2^ = 0.579), whereas the mean reconstructed page-level rating accounted for 9.9% (*R*^2^ = 0.099). These results support H2 while remaining specific to reconstructed rather than online page-level evaluations.

Analyses by task-length condition showed the same pattern in the long-task group (*N* = 16). Retrospective emotional valence correlated more strongly with PE (*r* = 0.822, *p* < 0.001) than with Average (*r* = 0.380, *p* = 0.147), and the difference was significant, Steiger’s *Z* = 2.70, *p* = 0.007, bootstrap 95% CI [0.034, 0.912]. PE explained 67.5% of the variance (*R*^2^ = 67.5%), whereas Average explained 14.4% (*R*^2^ = 14.4%).

In the short-task group (*N* = 16), retrospective emotional valence correlated with PE (*r* = 0.566, *p* = 0.022), but this correlation was not significantly larger than the correlation with Average (*r* = 0.217, *p* = 0.419), Steiger’s *Z* = 1.04, *p* = 0.297, bootstrap 95% CI [−0.423, 0.881]. PE explained 32.1% of the variance (*R*^2^ = 0.321), and Average explained 4.7% (*R*^2^ = 0.047). The wide bootstrap confidence interval indicates insufficient precision for estimating the magnitude of this difference in shorter tasks.

In summary, H2 was supported in the overall sample and in the long-task condition. While the descriptive pattern suggests that peak–end dominance may be less pronounced in shorter tasks, the small subgroup size precludes definitive moderation conclusions.

#### 3.1.4. Identification of Peak and End Interfaces

To identify the interfaces corresponding to peak and end experiences and to define targets for Study 2, page-level experience data were analyzed. The end experience was operationalized as the final stage of the task process; therefore, the end interface was defined as the final check-in page.

To identify operational targets for Study 2, page-level replay ratings were analyzed. The end page was defined structurally as the final check-in page. The candidate peak page was identified empirically from reconstructed page-level ratings in the long-task condition.

For the candidate peak page, we extracted the page on which each long-task participant reported the peak experience during page-level experience measurement. The frequency of peak experience reports was then tallied for each page. The task-completion page had the highest frequency (*n* = 6). The remaining 10 peak experience reports were dispersed across other pages, with no other page receiving more than 2 reports. Although the absolute count was small, the pattern indicated a relative concentration on the task-completion page rather than a uniform distribution. Given the small sample size and expected cell frequencies below five, Fisher’s exact test was used.

The task-completion page was therefore selected operationally as a theoretically plausible and empirically suggested candidate peak page for Study 2. A 2 × 2 contingency table was constructed with Page Category (candidate peak page vs. remaining pages) and Experience Type (peak experience vs. non-peak experience), and Fisher’s exact test was applied. As shown in Table 5, the distribution differed significantly (*p* < 0.001). The peak-report rate for the task-completion page was 37.5%, compared with an average rate of 3.47% for the remaining pages. This result supports the selection of the task-completion page as a reasonable intervention target in the present experiment. However, because the decision was based on six peak experience reports in a small subgroup, it should not be interpreted as validation of a universal peak page. Larger samples and online affect measures are needed to validate peak-page identification more strictly.

The task-completion page was operationally identified as a candidate peak-related interface within the present experimental procedure, and the check-in page was defined as the end page by task structure. These pages provided theoretically plausible and empirically suggested targets for the interface optimization design in Study 2.

### 3.2. Study 2

#### 3.2.1. Measuring Emotional Induction Efficacy and Selecting the Optimal Condition

**Figure 3:**
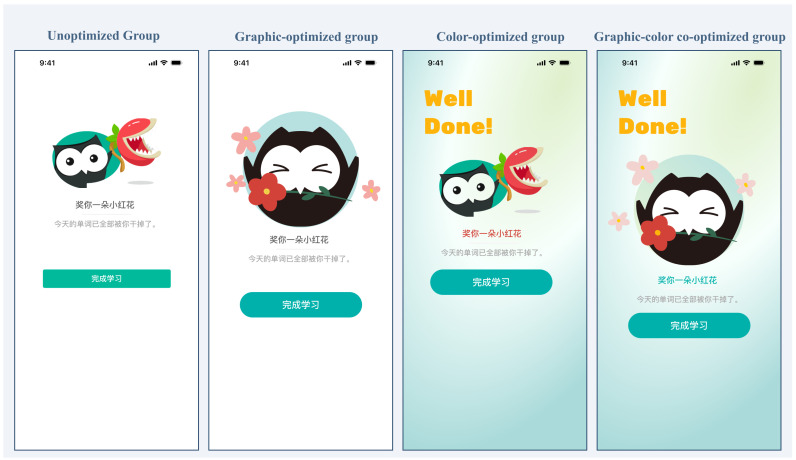
Peak-page interface designs under the four optimization conditions in Study 2. *Note.* The interface text is in Chinese, the language of the experimental materials. Key phrases: “奖你一朵小红花” = “Here’s a little red flower for you”; “今天的单词已全部被你干掉了” = “You’ve conquered all of today’s words!”; “完成学习” = “Complete Learning”.

**Figure 4:**
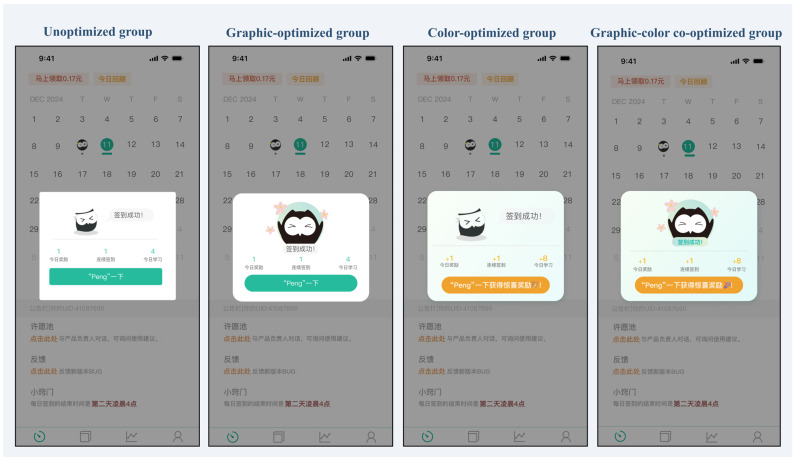
End-page interface designs under the four optimization conditions in Study 2. *Note.* The interface text is in Chinese, the language of the experimental materials. Key phrases — Top banner: “马上领取0.17元” = “Claim ¥0.17 Now”; “今日回顾” = “Today’s Review”. Pop-up card: “签到成功！” = “Check-in Successful!”; “今日奖励” = “Today’s Reward”; “连续签到” = “Consecutive Check-ins”; “今日学习” = “Today’s Study”; “Peng一下” = “Give it a Peng” (meaning ‘interact/tap’). Bottom section: “许愿池” = “Wishing Pool”; “反馈” = “Feedback”; “小窍门” = “Tips”; “每日签到的结果时间为第二天凌晨4点” = “Daily check-in results are released at 4:00 AM the following day”.

Study 2 retained the eight-word task because Study 1 yielded clearer peak–end evidence in the long-task condition. Targeting the task-completion page as a theoretically plausible and empirically suggested candidate peak page and the check-in page as the structurally defined end page, we created three optimized variants for each based on emotional design principles: a color-optimized version, an anthropomorphic-graphics-optimized version, and a combined color-plus-graphics version (Figure 3 and Figure 4).

To select the most effective variant, we conducted an independent preliminary rating study (*N* = 30). Participants evaluated all interfaces on smartphones in a quiet environment. Each interface was displayed for at least 5 s before the rating slider became active. Participants were instructed to imagine that they were completing a vocabulary-learning task and had reached the displayed page. They then rated each interface’s emotional valence using a VAS-based measure. The interface presentation was randomized within each page set.

An a priori G*Power analysis indicated a minimum sample size of 28 (power > 0.80). Thirty-three questionnaires were collected, and 30 were retained after screening. A 2 (Graphic: unoptimized vs. optimized) × 2 (Color: unoptimized vs. optimized) × 2 (Page: peak vs. end) repeated-measures ANOVA was conducted. Normality was approximately satisfied (all *p*s > 0.05), and sphericity was inherent for two-level factors.

**Figure 5:**
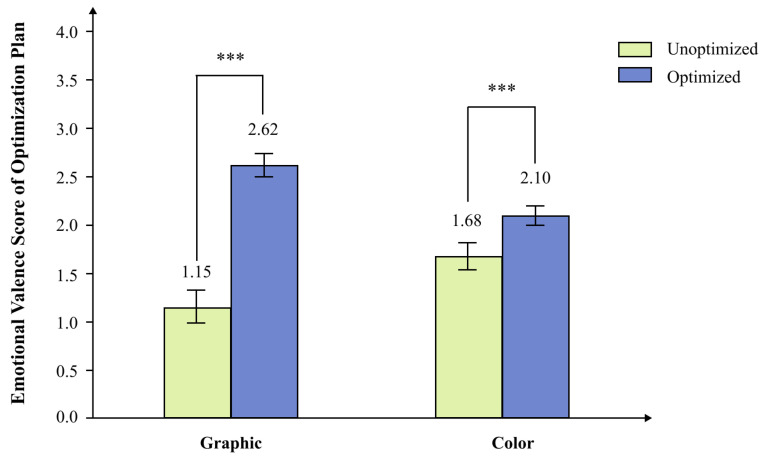
Emotional valence ratings across graphic-optimization and color-optimization conditions. *Note.* Values are estimated marginal means with standard-error bars. Exact means and standard errors are as follows: optimized graphic, *M* = 2.62, *SE* = 0.12; unoptimized graphic, *M* = 1.15, *SE* = 0.17; optimized color, *M* = 2.10, *SE* = 0.10; unoptimized color, *M* = 1.68, *SE* = 0.14. Asterisks indicate significance level: *** *p* < 0.001.

As shown in Figure 5, the main effect of the graphic was significant, *F*(1, 29) = 61.37, *p* < 0.001, *ηp*^2^ = 0.679, representing a very large effect. Ratings for the optimized graphic condition (*M* = 2.62, *SE* = 0.12) were significantly higher than ratings for the unoptimized graphic condition (*M* = 1.15, *SE* = 0.17), underscoring the pronounced emotional benefit of the anthropomorphic design in this rating task. The main effect of color was also significant, with *F*(1, 29) = 23.42, *p* < 0.001, *ηp*^2^ = 0.447, indicating a large effect. Ratings for the optimized color condition (*M* = 2.10, *SE* = 0.10) were significantly higher than ratings for the unoptimized color condition (*M* = 1.68, *SE* = 0.14), thereby confirming that color manipulation independently enhanced emotional valence.

**Figure 6:**
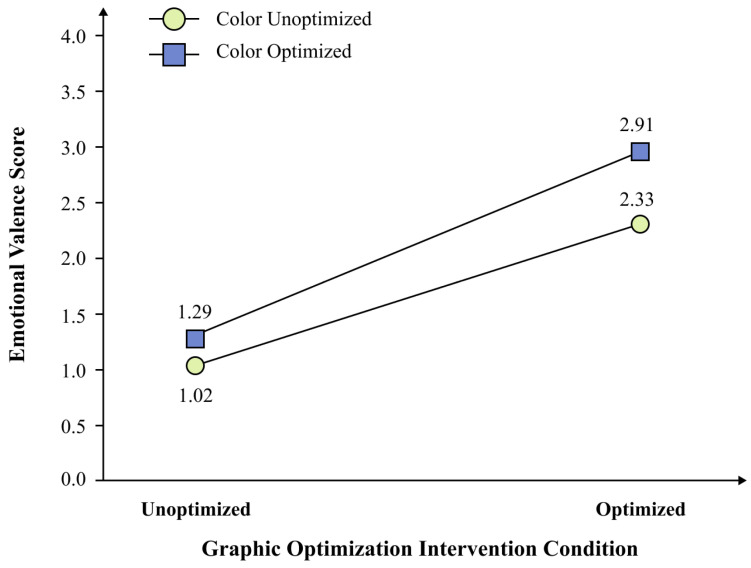
Interaction between graphic optimization and color optimization in the preliminary interface-rating study. *Note.* Values are estimated marginal means. The Graphic × Color interaction was significant, *F*(1, 29) = 7.58, *p* = 0.010, *ηp*^2^ = 0.207. Paired-samples *t*-tests showed that the color effect was larger under optimized graphics, *t*(29) = 5.49, *p* < 0.001, *d* = 1.00, than under unoptimized graphics, *t*(29) = 2.72, *p* = 0.011, *d* = 0.50. *N* = 30.

The Graphic × Color interaction was significant, *F*(1, 29) = 7.58, *p* = 0.010, *ηp*^2^ = 0.207 (Figure 6). Simple effect analyses with Bonferroni correction were conducted to decompose this interaction. The color effect was significant under optimized graphics, *t*(29) = 5.49, *p* < 0.001, Cohen’s *d* = 1.00, 95% CI [0.36, 0.79] (optimized graphic + optimized color: *M* = 2.91, *SE* = 0.10; optimized graphic + unoptimized color: *M* = 2.33, *SE* = 0.14). The color effect was also significant under unoptimized graphics but was smaller, *t*(29) = 2.72, *p* = 0.011, Cohen’s *d* = 0.50, 95% CI [0.07, 0.49] (unoptimized graphic + optimized color: *M* = 1.29, *SE* = 0.12; unoptimized graphic + unoptimized color: *M* = 1.02, *SE* = 0.17). This pattern suggests synergy between graphic and color optimization in the present rating task, but it was used only for stimulus selection.

The Graphic × Page interaction was also significant, *F*(1, 29) = 4.70, *p* = 0.039, *ηp*^2^ = 0.139. Graphic optimization increased ratings for both page types, but the increase was larger for the end page (peak page: optimized graphic *M* = 2.91, unoptimized graphic *M* = 1.73; end page: optimized graphic *M* = 2.33, unoptimized graphic *M* = 0.58). No other interactions were significant (*F*s < 2.72, *p*s > 0.110).

**Figure 7:**
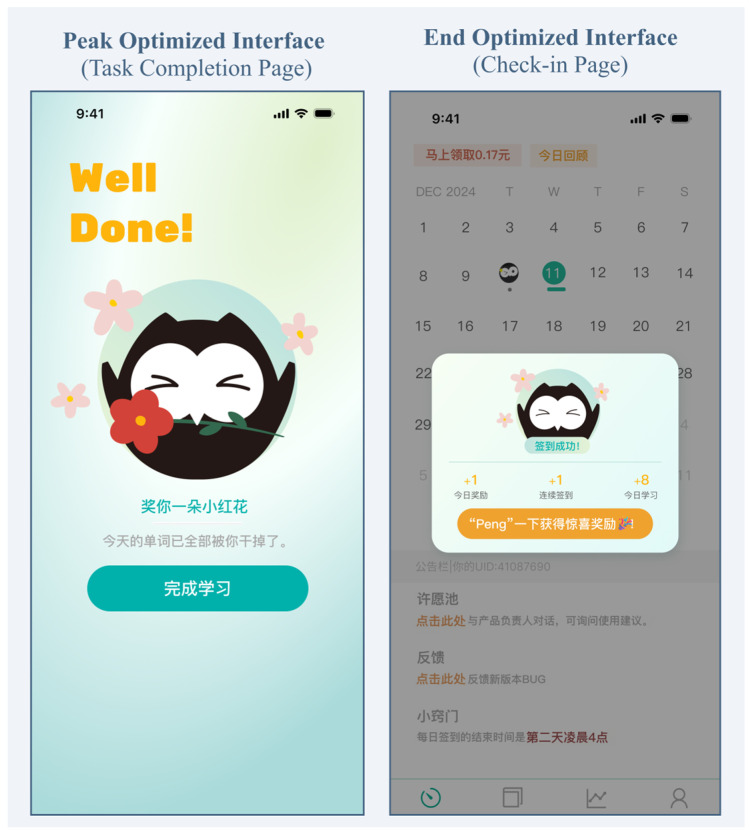
Final optimized versions of the peak-related page and the end page used in Study 2. *Note.* The interface text is in Chinese, the language of the experimental materials. For translations of all Chinese terms, see the note to Figure 3 and Figure 4.

To select the optimal scheme for the peak and end pages, we examined the estimated marginal means from the three-way interaction. For both pages, the highest-rated combination was graphic optimization plus color optimization (peak page: *M* = 3.14, *SE* = 0.10; end page: *M* = 2.69, *SE* = 0.15). Because the purpose of this preliminary test was strictly to confirm the emotional efficacy of the stimuli for the main experiment’s manipulation, this highest-rated combined condition was selected as the interface-optimization package. The interaction patterns informed material selection but did not generate the hypotheses (H3, H4, and RQ1), which were formulated a priori. Figure 7 shows the final optimized pages.

#### 3.2.2. Effects of Peak–End Interface Optimization on Retrospective Emotional Valence

Before analyzing retrospective emotional valence, we examined the effectiveness of the visual manipulation. Shapiro–Wilk normality tests indicated that the data in all groups were approximately normally distributed (*p*s > 0.05). Levene’s tests indicated unequal variances for both peak-page ratings (*F* = 24.872, *p* < 0.001) and end-page ratings (*F* = 8.962, *p* = 0.004); therefore, Welch *t*-tests were employed for between-group comparisons (Table 6).

Welch *t*-tests showed that the peak-optimized group rated the peak page significantly higher (*M* = 3.350, *SD* = 0.436) than the non-peak-optimized group (*M* = 2.063, *SD* = 1.111), *t*(35.1) = 5.707, *p* < 0.001, Cohen’s *d* = 1.53. The end-optimized group rated the end page significantly higher (*M* = 2.821, *SD* = 0.714) than the non-end-optimized group (*M* = 1.677, *SD* = 1.366), *t*(40.7) = 3.925, *p* < 0.001, Cohen’s *d* = 1.049. These results confirm that both visual manipulations were successfully implemented in the main experiment.

To examine the effects of peak optimization and end optimization on retrospective emotional valence, a two-way ANOVA with factors 2 (Peak optimization: unoptimized vs. optimized) × 2 (End optimization: unoptimized vs. optimized) was conducted using data from 56 participants. Shapiro–Wilk tests indicated that retrospective emotional valence ratings were normally distributed in each group (*p* > 0.05). Levene’s test indicated homogeneous variances across groups (*p* > 0.05), satisfying the assumptions for ANOVA.

Across the four conditions, mean retrospective emotional valence ratings were as follows: non-optimized peak and non-optimized end group (*M* = 1.60, *SD* = 0.63), optimized peak and non-optimized end group (*M* = 2.60, *SD* = 0.85), non-optimized peak and optimized end group (*M* = 2.69, *SD* = 0.51), and optimized peak and optimized end group (*M* = 2.87, *SD* = 0.60), with 14 participants in each group. Marginal means showed that participants who received peak optimization (*M* = 2.74, *SD* = 0.73) reported higher retrospective emotional valence than those who did not receive peak optimization (*M* = 2.14, *SD* = 0.79). Similarly, participants who received end optimization (*M* = 2.78, *SD* = 0.56) reported higher retrospective emotional valence than those who did not receive end optimization (*M* = 2.10, *SD* = 0.89).

**Figure 8:**
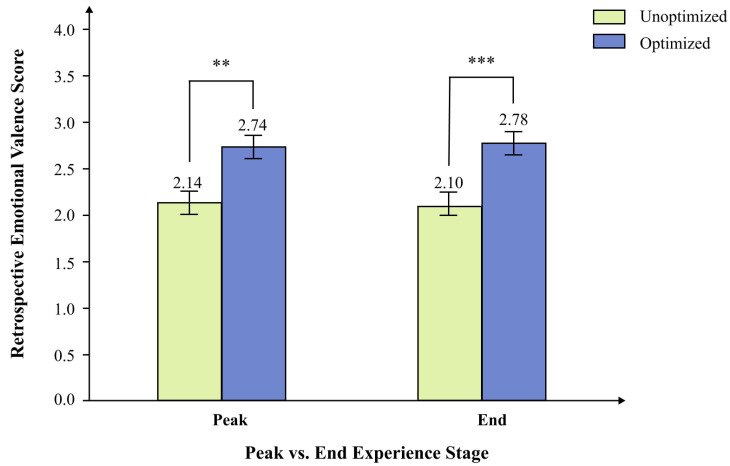
Main effects of peak optimization and end optimization on retrospective emotional valence. *Note.* Values are estimated marginal means with standard-error bars. The main effect of peak optimization was significant, *F*(1, 52) = 11.27, *p* = 0.001, *ηp*^2^ = 0.18 (optimized: *M* = 2.74, *SE* = 0.14; unoptimized: *M* = 2.14, *SE* = 0.15). The main effect of end optimization was significant, *F*(1, 52) = 14.87, *p* < 0.001, *ηp*^2^ = 0.22 (optimized: *M* = 2.78, *SE* = 0.11; unoptimized: *M* = 2.10, *SE* = 0.17). Asterisks indicate the significance level of the corresponding pairwise comparison: ** *p* < 0.01, *** *p* < 0.001.

As shown in Figure 8, the ANOVA results revealed a significant main effect of peak optimization, *F*(1, 52) = 11.27, *p* = 0.001, *ηp*^2^ = 0.18, a large effect indicating that peak optimization was associated with higher retrospective emotional valence ratings in the present experiment, supporting hypothesis H3. The main effect of end optimization was also significant, *F*(1, 52) = 14.87, *p* < 0.001, *ηp*^2^ = 0.22, a large effect size indicating that end optimization was likewise associated with higher retrospective emotional valence ratings in the present experiment, supporting hypothesis H4.

**Figure 9:**
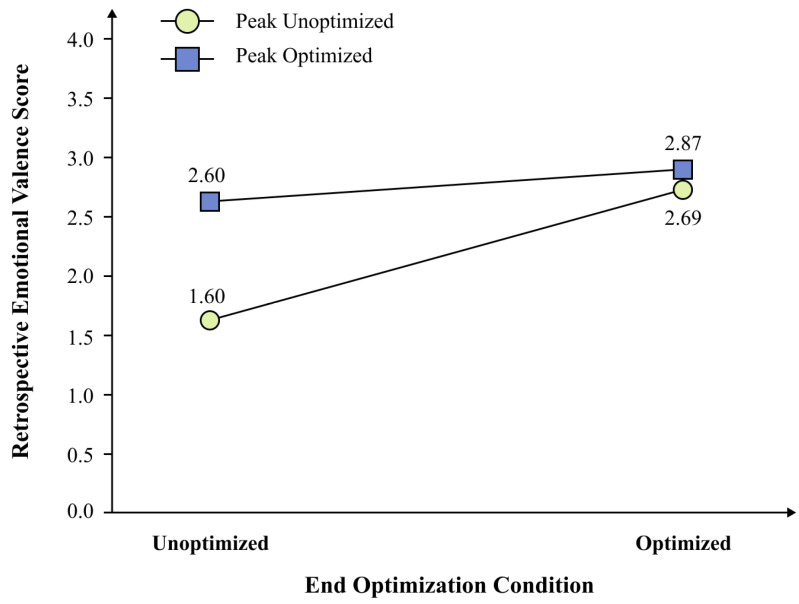
Interaction between peak optimization and end optimization on retrospective emotional valence. *Note.* Values are estimated marginal means. The interaction was significant, *F*(1, 52) = 5.24, *p* = 0.026, *ηp*^2^ = 0.09. Group means (SEs in parentheses) were as follows: non-optimized peak and non-optimized end, *M* = 1.60 (0.17); optimized peak and non-optimized end, *M* = 2.60 (0.23); non-optimized peak and optimized end, *M* = 2.69 (0.14); optimized peak and optimized end, M = 2.87 (0.16). Simple effect analyses with Bonferroni correction revealed a compensatory pattern: each optimization significantly improved valence only when the other was absent (*ts*(26) ≥ 3.53, *p*_Bonf_ ≤ 0.003, *ds* ≥ 1.33). *N* = 56.

The interaction between peak optimization and end optimization was significant (Figure 9), *F*(1, 52) = 5.24, *p* = 0.026, *ηp*^2^ = 0.09, a medium effect size. To decompose this interaction, simple effect analyses with Bonferroni correction were conducted. The simple effect of peak optimization was significant when the end page was unoptimized, *t*(26) = 3.53, *p*_Bonf_ = 0.003, Cohen’s *d* = 1.33, but was not significant when the end page was already optimized, *t*(26) = 0.89, *p*_Bonf_ = 0.763, *d* = 0.34. Similarly, the simple effect of end optimization was significant when the peak page was unoptimized, *t*(26) = 4.98, *p*_Bonf_ < 0.001, *d* = 1.88, but was not significant when the peak page was already optimized, *t*(26) = 0.99, *p*_Bonf_ = 0.658, *d* = 0.38. Bonferroni-corrected post hoc pairwise comparisons across the four groups (Table 7) confirmed that the non-optimized condition differed significantly from each of the three optimized conditions, whereas the three optimized conditions did not differ significantly from one another. These results are consistent with a non-additive interaction pattern in which the incremental benefit of optimizing one node was substantially reduced when the other node was already optimized.

#### 3.2.3. Behavioral Outcome: Word-Learning Accuracy

To examine whether peak and end optimizations influenced learning performance, a 2 (Peak optimization: unoptimized vs. optimized) × 2 (End optimization: unoptimized vs. optimized) between-subjects ANOVA was conducted on word-learning accuracy.

**Figure 10:**
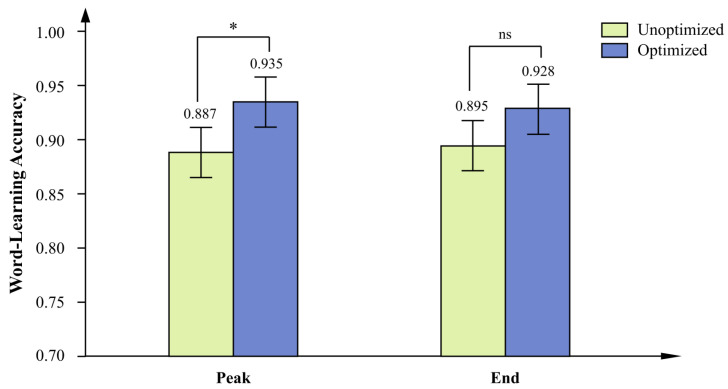
Main effects of peak optimization and end optimization on word-learning accuracy. *Note.* Values are estimated marginal means with standard-error bars. The main effect of peak optimization was significant, *F*(1, 52) = 4.44, *p* = 0.040, *ηp*^2^ = 0.079 (optimized: *M* = 0.935, *SE* = 0.013; unoptimized: M = 0.887, SE = 0.019). The main effect of end optimization was not significant, *F*(1, 52) = 2.09, *p* = 0.154, *ηp*^2^ = 0.039 (optimized: *M* = 0.928, *SE* = 0.016; unoptimized: *M* = 0.895, *SE* = 0.016). ns = not significant. *N* = 56. Asterisks indicate the significance level of the corresponding pairwise comparison: * *p* < 0.05.

**Figure 11:**
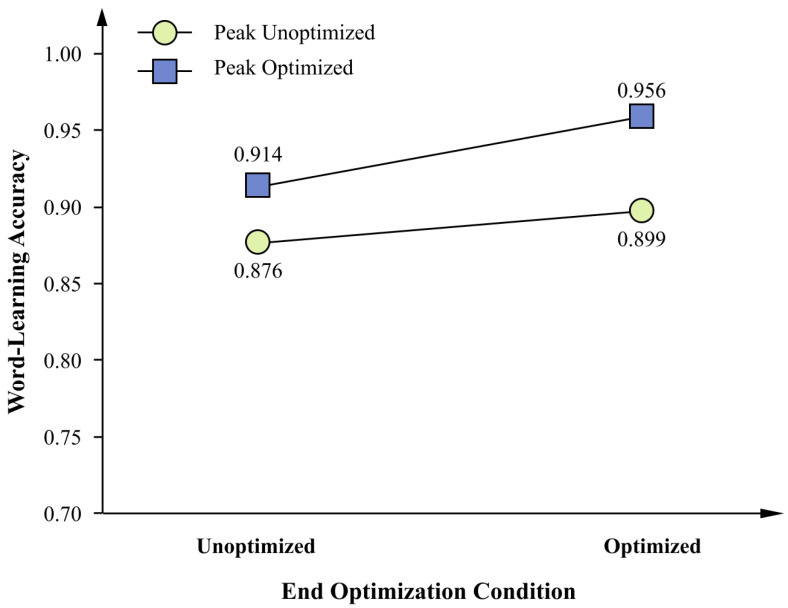
Interaction between peak optimization and end optimization on word-learning accuracy. *Note.* Values are estimated marginal means. The interaction between peak optimization and end optimization was not significant, *F*(1, 52) = 0.17, *p* = 0.684, *ηp*^2^ = 0.003, and should not be interpreted. Group means (*SE*s in parentheses) were as follows: non-optimized peak and non-optimized end, *M* = 0.876 (0.024); optimized peak and non-optimized end, M = 0.914 (0.021); non-optimized peak and optimized end, *M* = 0.899 (0.029); optimized peak and optimized end, *M* = 0.956 (0.013). *N* = 56. ns = Not significant.

The ANOVA revealed a significant main effect of peak optimization (Figure 10), *F*(1, 52) = 4.44, *p* = 0.040, *ηp*^2^ = 0.079, and a medium effect size, indicating that, collapsing across end-optimization conditions, accuracy was higher when the peak page was optimized (*M* = 0.935, *SD* = 0.068) than when it was not optimized (*M* = 0.887, *SD* = 0.099). The main effect of end optimization was not significant, *F*(1, 52) = 2.09, *p* = 0.154, *ηp*^2^ = 0.039, and the interaction was also not significant (Figure 11), *F*(1, 52) = 0.17, *p* = 0.684, *ηp*^2^ = 0.003. Collapsed across peak-optimization conditions, accuracy was descriptively higher when the end page was optimized (*M* = 0.928, *SD* = 0.084) than when it was not optimized (*M* = 0.895, *SD* = 0.086).

Bonferroni-corrected post hoc comparisons were conducted across the four groups (Table 8). The only statistically reliable difference among individual conditions was between the fully optimized group and the non-optimized control group, *M*_diff_ = 0.081, *p*_Bonf_ = 0.041. The large effect size (Cohen’s *d* = 1.11) indicates a substantial standardized difference between these two conditions relative to the observed variability.

These results indicate that only the combination of peak and end optimization produced a statistically reliable improvement in word-learning accuracy relative to the no-optimization baseline. Optimizing either selected page in isolation did not yield a significant gain after correction for multiple comparisons.

## 4. Discussion

This research examined whether peak–end patterns appear in a brief controlled vocabulary-learning task and whether visual optimization at selected completion and check-in pages improves retrospective emotional valence. Across two experiments, the findings were broadly consistent with the peak–end rule, but they should be interpreted within the constraints of a short laboratory task and reconstructed affect ratings.

### 4.1. Evidence for the Peak–End Effect and the Contextual Role of Task Length

Study 1 produced findings broadly consistent with the peak–end rule. Although the long-task group spent significantly more time on the task, retrospective emotional valence did not differ between groups. This null difference is consistent with duration neglect under the present short task–episode manipulation. However, because word count also changed cognitive load, exposure frequency, and task progression, the result cannot be interpreted as strong evidence for pure temporal duration neglect. Low statistical power, insufficient temporal contrast, compensatory effects between fatigue and achievement, and characteristics of the selected vocabulary items remain plausible alternative explanations.

Steiger’s *Z* tests showed that retrospective emotional valence was significantly more strongly associated with the peak–end average than with the mean of reconstructed page-level ratings. In the overall sample, the peak–end average accounted for 57.9% of the variance (*R*^2^ = 0.579), whereas the mean reconstructed page-level rating explained 9.9% (*R*^2^ = 0.099). This indicates that, under the present conditions, the reconstructed peak–end summary was a stronger predictor of retrospective evaluation than the average of all reconstructed page-level ratings. This finding is consistent with the theoretical claim that representative moments receive disproportionate weight (Ariely & Carmon, 2000) and with the availability heuristic (Tversky & Kahneman, 1973). However, alternative interpretations must be acknowledged: the prominence of the end moment could partly reflect a recency effect, and the weaker association with mean reconstructed page-level ratings could reflect general memory decay rather than a specific peak–end heuristic. The present design therefore supports, but does not fully isolate, the peak–end mechanism.

Regarding the contextual role of task length, descriptive patterns in *R*^2^ and effect sizes suggest that peak–end dominance may be more pronounced in longer tasks, but this moderating effect could not be definitively established. The wide confidence interval for the correlation difference in the short-task group (−0.42 to 0.88) reflects limited estimation precision due to the small sample size. Consequently, while the data provide descriptive hints that the average experience may retain some relative weight in shorter tasks, claims about task length qualifying the peak–end effect must remain exploratory and require verification with studies powered to detect moderation.

### 4.2. Effects of Visual Design Elements on Interface Emotional Valence

The emotion induction experiment in Study 2 showed that both color optimization and anthropomorphic graphic optimization significantly increased interface emotional valence ratings, with large effect sizes. This finding extends color psychology (Elliot & Maier, 2014) and anthropomorphism theory (Epley et al., 2007) to mobile interactive interfaces, showing that even brief interface exposure can influence users’ emotional experiences. The effect of graphic optimization (*ηp*^2^ = 0.679) was larger than that of color optimization (*ηp*^2^ = 0.447), suggesting that anthropomorphic cues may be a particularly potent trigger of positive affect in this context. This interpretation is consistent with media equation theory, which proposes that users apply social rules to computer interfaces and respond to social cues such as characters and facial expressions (Reeves & Nass, 1996).

The Graphic × Color interaction indicated that the combined visual package produced higher valence ratings than either element alone in the preliminary rating task. A synergy account is plausible because color and social-agency cues may reinforce one another (Sundar et al., 2016). However, this account was not tested directly, and the interaction was used only to select the stimulus package for Study 2. Similarly, the larger graphic effect on the end page may reflect a lower baseline for that page rather than a general property of end moments.

### 4.3. Effects of Peak–End Interface Optimization on Retrospective Emotional Valence and Their Interaction

Study 2 showed that both peak optimization and end optimization independently increased retrospective emotional valence, with significant main effects and medium-to-large effect sizes. Critically, the significant Peak × End interaction (addressing RQ1) revealed a non-additive pattern: the benefit of optimizing one node was larger when the other was unoptimized, and substantially smaller when the other was already optimized. Thus, the two optimizations did not simply add together; instead, their effects were interdependent, and the presence of one optimization partially attenuated the additional impact of the other.

This finding extends discussions of the peak–end rule in interface-design contexts. While prior studies examined how peak and end moments predict retrospective evaluation, our experiment tested how optimizing specific pages identified as a candidate peak and the end affect overall judgments. The results suggest that the benefits of these page-level optimizations on retrospective evaluation may not be strictly additive and may depend on the optimization state of the other page.

Two post hoc accounts are compatible with this pattern and may guide future research. First, emotional appraisal may be subject to a ceiling effect: once one selected page reaches a highly positive level, the perceptual impact of further improvement at the other page may be reduced. Second, retrospective evaluation may have limited capacity to integrate multiple positive cues, so a second cue may add less incremental value after a dominant positive anchor has formed. These accounts are hypotheses for future research rather than conclusions from the present data. Future studies should test them using nonlinear modeling, threshold designs, online affect measures, or psychophysiological measures.

The present study also examined whether interface optimization influenced objective word-learning accuracy. Although peak optimization showed a significant main effect, only the combined peak-and-end condition significantly outperformed the non-optimized control condition after correction for multiple comparisons (*p* = 0.041, *d* = 1.11). Neither single-node condition produced a statistically reliable corrected gain. This learning-accuracy result is considered only a preliminary finding, and future research should further incorporate delayed retention measures, baseline vocabulary knowledge, and process indicators such as cognitive effort, attention, motivation, and arousal.

In summary, the data show that optimization of the selected completion and check-in pages improved retrospective valence and produced a non-additive interaction pattern. The fully optimized condition also improved word-learning accuracy relative to the baseline, whereas single-page optimizations did not show reliable corrected gains. These findings support the practical value of targeted page optimization in this short controlled task, but they do not establish a general mechanism for learning improvement.

### 4.4. Theoretical Contributions and Practical Implications

This study makes three theoretical contributions. First, it provides preliminary evidence that peak–end patterns can be observed in a brief vocabulary-learning interaction involving semantic processing. Second, it shows that retrospective valence was more strongly associated with reconstructed peak–end summaries than with the mean of reconstructed page-level ratings. Third, it identifies a non-additive interaction between peak and end optimization, suggesting that the two key moments contribute to retrospective evaluation in an interdependent rather than strictly additive manner.

These findings suggest several design implications for vocabulary-learning applications and similar short digital learning tasks. Designers may first identify candidate peak-related and end moments in the user journey and then test whether targeted optimization of these selected pages improves remembered experience. Among the visual elements tested, anthropomorphic graphics produced a larger valence enhancement than color optimization, and their combination yielded the highest ratings within the present design space. However, these effect sizes were obtained from a specific interface set, a student sample, a single app genre, and a short-term task. The present data, therefore, indicate that anthropomorphic cues can be effective in such contexts but do not yet support generalized prioritization of one element over the other across all learning applications. Furthermore, because the two optimizations partially substituted for one another, allocating resources to a single key node can still substantially improve experience. Within the present controlled task, combined peak-and-end optimization was the most promising condition because it was the only condition that significantly improved word-learning accuracy relative to the baseline.

### 4.5. Limitations and Future Directions

This study has several limitations that warrant further examination in future research.

First, regarding sample limitations, although meeting a priori power thresholds for detecting medium effects, the small sample sizes (Study 1: *N* = 32; Study 2: *N* = 56) limited statistical power for detecting small effects and constrained generalizability. Furthermore, the homogeneous sample of Chinese undergraduates restricts demographic and cultural generalizability. Finally, individual differences, such as baseline learning ability, prior knowledge, and emotional sensitivity, were not measured. While such individual differences are inevitable, and our use of rigorous random assignment mitigated their confounding effects, these traits may still moderate the peak–end rule. Future research should use larger and more diverse samples and include these individual differences as covariates.

Second, concerning internal validity and manipulation confounds, manipulating task length via word count inevitably confounded temporal duration with cognitive load and exposure frequency. Because no direct cognitive load measure was included (e.g., NASA-TLX), the pure effect of duration cannot be isolated, and the null result may reflect the cancellation of competing emotions or insufficient power to detect small effects. Additionally, the manipulation check relied on objective completion time rather than perceived duration. Future research should independently manipulate duration and cognitive load, measure perceived duration, and include interference tasks or manipulate the temporal placement of peak and end moments to dissociate the peak–end rule from recency effects and memory bias.

Third, emotions were measured primarily through retrospective self-reports. Page-level ratings obtained through task replay captured reconstructed affect rather than genuine online experience. Furthermore, this study measured only valence; although prior research suggests that retrospective evaluations are primarily driven by the valence of peak and end moments, the potential moderating role of arousal cannot be ruled out. Finally, the two independent studies used different valence scales, which preclude direct quantitative comparison of absolute valence scores across studies. Future studies should employ non-intrusive physiological and behavioral measures, such as skin conductance, facial expression analysis, eye-tracking, or continuous affect ratings, to better approximate online affective dynamics; they should also include arousal dimensions and maintain scale consistency across studies.

Fourth, this study examined only color and anthropomorphic graphics and did not test other design elements, such as typography or motion effects. Moreover, although visual optimization at selected pages (the candidate peak-related page and end page) improved retrospective emotional valence and fully combined optimization improved word-learning accuracy, it remains unclear whether these manipulations altered perceived usability or cognitive load. Future research should test a broader range of design variables and evaluate the functional consequences of emotional nudges for both user experience and learning efficacy.

Fifth, although the artificial and highly controlled vocabulary task (learning four or eight isolated words) successfully manipulated a significant difference in objective duration, it cannot fully replicate the complexity and self-regulated nature of real-world digital learning. The short absolute duration may restrict the emergence of more pronounced duration-neglect effects. Additionally, the study lacked follow-up assessments, so the persistence of optimization effects remains unknown. Future research should extend task lengths to simulate natural learning sessions and adopt longitudinal designs to evaluate long-term effects on retention, user motivation, and actual learning behavior.

## 5. Conclusions

Study 1 found that increasing the word count from four to eight prolonged task completion time without significantly altering retrospective valence. This result is consistent with duration neglect under a short task–episode manipulation, but it is not a strong test of pure temporal duration neglect because word count also changed cognitive load, exposure frequency, and task progression. Retrospective valence was more strongly associated with the peak–end average (*r* = 0.761) than with the mean of reconstructed page-level ratings (*r* = 0.314; Steiger’s *Z* = 3.03, *p* = 0.002). This finding provides preliminary evidence that retrospective evaluations in the present brief vocabulary-learning task were better aligned with reconstructed peak–end summaries than with average reconstructed page-level ratings.

Study 2 showed that visual optimization of the theoretically plausible and empirically suggested candidate peak page and the structurally defined end page each improved retrospective emotional valence under controlled conditions. Their interaction was non-additive (*ηp*^2^ = 0.09): the incremental benefit of optimizing one selected page diminished when the other selected page was already optimized. For word-learning accuracy, only the combined peak-and-end condition significantly outperformed the non-optimized control after correction (*p* = 0.041, *d* = 1.11); neither single-page optimization yielded a reliable corrected gain.

Overall, this study provides preliminary evidence that retrospective evaluations in a brief controlled vocabulary-learning task are more closely aligned with peak–end summaries than with average reconstructed page-level ratings, and that visual optimization of the selected completion page (candidate peak-related page) and check-in page (end page) can improve retrospective valence under controlled conditions. These findings are not intended to establish a universal peak page, a pure duration-neglect effect, or a stable mechanism for learning improvement. Future research should test longer and more naturalistic learning sessions, measure online affect and cognitive load, and examine whether the effects generalize across learner populations and app genres.

## Figures and Tables

**Table 1:** Descriptive statistics and internal consistency reliability of word recognition difficulty across CEFR levels (*N* = 30).

CEFR Levels	Word Count	*M*	*SD*	Cronbach’s *α*
B1	10	2.95	1.02	0.89
B2	10	4.14	1.69	0.95
C1	10	5.03	1.54	0.94
C2	10	5.91	1.32	0.91

*Note.* Perceived difficulty was measured on a 7-point Likert scale (1 = very easy, 7 = very difficult). Cronbach’s *α* values exceeded 0.70 for all levels, indicating good internal consistency.

**Table 2:** Descriptive statistics and Welch’s *t*-test results for task completion time by group.

Groups	*n*	*M* (Seconds)	*SD* (Seconds)	Levene *p*	Welch *t*	*df*	*p*	Cohen’s *d*
Short-task group	16	60.50	21.48	0.002	−4.23	21.06	<0.001	−1.50
Long-task group	16	114.94	46.79					

*Note.* Data were normally distributed. Welch’s *t*-test was used because the homogeneity-of-variance assumption was violated. Task completion time was significantly shorter in the short-task group than in the long-task group (*p* < 0.001), confirming successful experimental manipulation.

**Table 3:** Descriptive statistics and Welch’s *t*-test results for retrospective emotional valence by group.

Groups	*n*	*M*	*SD*	Levene *p*	Welch *t*	*df*	*p*	Cohen’s *d*
Short-task group	16	7.00	0.89	0.020	0.75	25.49	0.459	0.27
Long-task group	16	6.69	1.40					

*Note.* The data were normally distributed. Because homogeneity of variances was violated (*p* = 0.020), Welch’s *t*-test was used. No significant difference was found between groups (*p* = 0.459).

**Table 4:** Comparison of correlations between retrospective emotional valence and peak–end average versus mean of reconstructed page-level ratings.

Condition	Predictor	*r*	*p*	Steiger’s *Z*	*p*	95% CI for Difference (Bootstrap)
Overall Sample (*N* = 32)	Peak–end average (PE)	0.761	<0.001	3.03	0.002	[0.147, 0.747]
	Mean of reconstructed page-level ratings	0.314	0.080			
Long-task group (*n* = 16)	Peak–end average (PE)	0.822	<0.001	2.70	0.007	[0.034, 0.912]
	Mean of reconstructed page-level ratings	0.380	0.147			
Short-task group (*n* = 16)	Peak–end average (PE)	0.566	0.022	1.04	0.297	[−0.423, 0.881]
	Mean of reconstructed page-level ratings	0.217	0.419			

*Note*. PE = peak–end average; Average = mean of reconstructed page-level ratings. Steiger’s *Z* tests compared dependent correlation coefficients (PE vs. Average) within each condition. Bootstrap 95% confidence intervals for correlation differences were based on 10,000 resamples.

**Table 5:** Contingency table of peak experience report frequency by page category.

Page Category	Peak Experience	Non-Peak Experience	Total
Candidate peak page (task-completion page)	6	10	16
Remaining Pages	10	278	288
Total	16	288	304

*Note.* Fisher’s exact test showed a significant difference in reconstructed peak experience distribution between the assumed peak page and other pages (*p* < 0.001).

**Table 6:** Manipulation check: Comparison of page ratings by condition.

Outcome Variable	Condition	*n*	*M*	*SD*	*t*	*df*	*p*	Cohen’s *d*
Peak-page rating	Peak-optimized	28	3.350	0.436	5.707	35.1	<0.001	1.53
	Non-peak-optimized	28	2.063	1.111				
End-page rating	End-optimized	28	2.821	0.714	3.925	40.7	<0.001	1.049
	Non-end-optimized	28	1.677	1.366				

*Note.* Levene’s test indicated unequal variances for both peak-page ratings (*F* = 24.872, *p* < 0.001) and end-page ratings (*F* = 8.962, *p* = 0.004); therefore, Welch *t*-tests were used. Cohen’s *d* was calculated using the pooled standard deviation. All groups had equal sample sizes (*n* = 28).

**Table 7:** Post hoc pairwise comparisons for retrospective emotional valence across optimization conditions.

Comparison	*M*_diff_	*t*	*p*	*p*_Bonf_	Cohen’s *d*
Control vs. End-only	1.082	4.983	<0.001	<0.001 ***	1.883
Control vs. Peak-only	0.994	3.526	0.002	0.010 *	1.333
Control vs. Fully optimized	1.270	5.469	<0.001	<0.001 ***	2.067
End-only vs. Peak-only	−0.088	−0.332	0.743	1.000	0.125
End-only vs. Fully optimized	0.188	0.891	0.381	1.000	0.337
Peak-only vs. Fully optimized	0.276	0.994	0.329	1.000	0.376

*Note. M*_diff_ = mean difference (latter group − former group). Control = non-optimized peak and non-optimized end; End-only = non-optimized peak and optimized end; Peak-only = optimized peak and non-optimized end; Fully optimized = optimized peak and optimized end. *p*_Bonf_ = Bonferroni-corrected *p* value adjusted for six comparisons. * *p*_Bonf_ < 0.05, *** *p*_Bonf_ < 0.001.

**Table 8:** Post hoc pairwise comparisons for word-learning accuracy across optimization conditions.

Comparison	*M*_diff_	*t*	*p*	*p*_Bonf_	Cohen’s *d*
Control vs. End-only	0.024	0.622	0.539	1.000	0.24
Control vs. Peak-only	0.039	1.201	0.241	1.000	0.45
Control vs. Fully optimized	0.081	2.943	0.007	0.041 *	1.11
End-only vs. Peak-only	0.015	0.414	0.682	1.000	0.16
End-only vs. Fully optimized	0.057	1.778	0.087	0.523	0.67
Peak-only vs. Fully optimized	0.042	1.682	0.105	0.627	0.64

*Note. M*_diff_ = mean difference (latter group − former group). Control = non-optimized peak and non-optimized end; End-only = non-optimized peak and optimized end; Peak-only = optimized peak and non-optimized end; Fully optimized = optimized peak and optimized end. *p*_Bonf_ = Bonferroni-corrected *p* value (raw *p* × 6). * *p*_Bonf_ < 0.05.

## Data Availability

The datasets, selected stimuli, interface materials, and analysis scripts supporting the findings of this study are available from the corresponding author upon reasonable request, subject to participant privacy protections and institutional ethics restrictions.

## Abbreviations

The following abbreviations are used in this manuscript:

ANOVA	Analysis of Variance
CEFR	Common European Framework of Reference for Languages
EMM	Estimated Marginal Means
HCI	Human–Computer Interaction
MET	Media Equation Theory
PE	Peak–End Average/Peak–End Rule
SAM	Self-Assessment Manikin
UX	User Experience
VAS	Visual Analog Scale

## Appendix A. SAM Emotion Rating Scale

**Rating**									
Graphic									
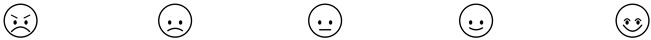
Item	1	2	3	4	5	6	7	8	9
valence									

## Appendix B. VAS-Based Continuous Emotional Valence Rating Scale

*Note.* The blue dot indicates the draggable slider handle; participants moved it left or right along the scale to register their rating. The cursor icon reflects the interactive nature of the online instrument.
